# 4-Chloro­anilinium thio­cyanate

**DOI:** 10.1107/S160053681202377X

**Published:** 2012-06-02

**Authors:** Siti Fairus M. Yusoff, F. Salem Halima, Bohari M. Yamin

**Affiliations:** a School of Chemical Sciences and Food Technology, Universiti Kebangsaan Malaysia, 43600 Bangi, Selangor, Malaysia

## Abstract

In the title compound, C_6_H_7_ClN^+^·NCS^−^, the benzene ring and the protonated amine and chloro substituents are nearly planar, with a maximum deviation of 0.002 (2) Å for the N atom. In the crystal, the mol­ecules are linked by N—H⋯N and N—H⋯S hydrogen bonds into a chain along the *b* axis.

## Related literature

For bond-length data see: Allen *et al.* (1987 ▶) and for a description of the Cambridge Structural Database, see: Allen (2002 ▶). For related thio­cyanate structures, see: Salem *et al.* (2012 ▶); Selvakumaran *et al.* (2011 ▶); Khawar Rauf *et al.* (2008 ▶). 
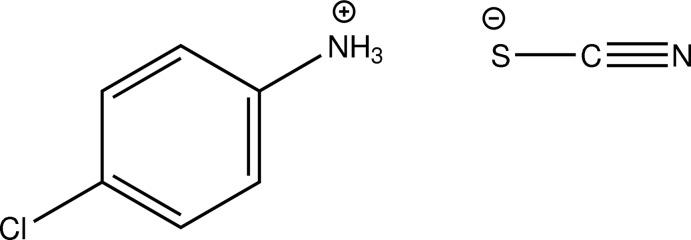


## Experimental

### 

#### Crystal data

C_6_H_7_ClN^+^·NCS^−^
*M* *_r_* = 186.66
Orthorhombic, 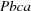

*a* = 7.743 (2) Å
*b* = 7.199 (2) Å
*c* = 31.913 (10) Å
*V* = 1778.8 (10) Å^3^
*Z* = 8
Mo *K*α radiation
μ = 0.60 mm^−1^
*T* = 298 K
0.50 × 0.43 × 0.30 mm

#### Data collection

Bruker SMART APEX CCD area-detector diffractometer
Absorption correction: multi-scan (*SADABS*; Bruker, 2000 ▶) *T*_min_ = 0.754, *T*_max_ = 0.841
10422 measured reflections
1846 independent reflections
1628 reflections with *I* > 2σ(*I*)
*R*_int_ = 0.024

#### Refinement

*R*[*F* ^2^ > 2σ(*F* ^2^)] = 0.039
*wR*(*F* ^2^) = 0.108
*S* = 1.18
1846 reflections
112 parameters
3 restraints
H atoms treated by a mixture of independent and constrained refinement
Δρ_max_ = 0.31 e Å^−3^
Δρ_min_ = −0.19 e Å^−3^

### 

Data collection: *SMART* (Bruker, 2000 ▶); cell refinement: *SAINT* (Bruker, 2000 ▶); data reduction: *SAINT*; program(s) used to solve structure: *SHELXS97* (Sheldrick, 2008 ▶); program(s) used to refine structure: *SHELXL97* (Sheldrick, 2008 ▶); molecular graphics: *SHELXTL* (Sheldrick, 2008 ▶); software used to prepare material for publication: *SHELXTL*, *PARST* (Nardelli, 1995 ▶) and *PLATON* (Spek, 2009 ▶).

## Supplementary Material

Crystal structure: contains datablock(s) global, I. DOI: 10.1107/S160053681202377X/bq2362sup1.cif

Structure factors: contains datablock(s) I. DOI: 10.1107/S160053681202377X/bq2362Isup2.hkl

Supplementary material file. DOI: 10.1107/S160053681202377X/bq2362Isup3.cml

Additional supplementary materials:  crystallographic information; 3D view; checkCIF report

## Figures and Tables

**Table 1:** Hydrogen-bond geometry (Å, °)

*D*—H⋯*A*	*D*—H	H⋯*A*	*D*⋯*A*	*D*—H⋯*A*
N1—H1*A*⋯N2^i^	0.87 (2)	2.03 (1)	2.888 (2)	172 (2)
N1—H1*B*⋯N2^ii^	0.86 (1)	2.08 (1)	2.911 (3)	162 (2)
N1—H1*C*⋯S1^iii^	0.87 (2)	2.48 (3)	3.285 (2)	155 (2)

Symmetry codes: (i) 
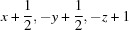
; (ii) 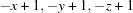; (iii) 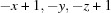.

**Fig. 1.**
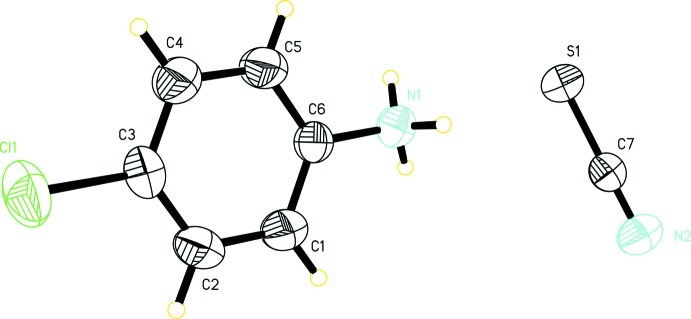
: Molecular structure of the title compound, (I), with 50% probability displacement ellipsoids.

**Fig. 2.**
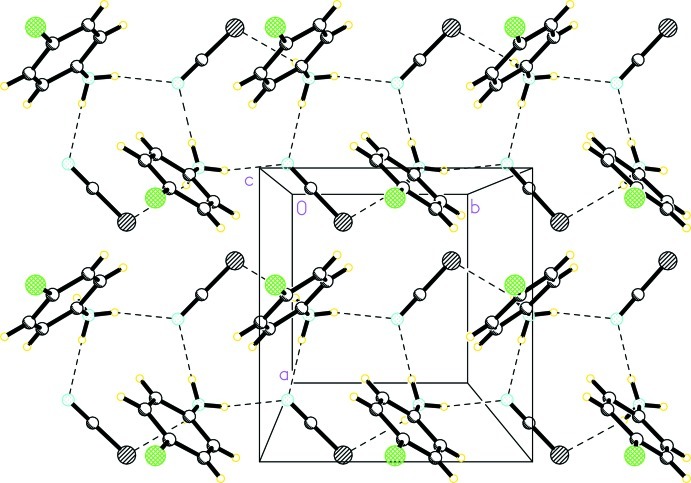
: Packing diagram of (I), viewed down b axis. The dashed lines denote hydrogen bonds.

## supplementary crystallographic information

### Comment

The title compound (Fig. 1) is an organic thiocyanate similar to dicylohexylammonium thiocyanate (Khawar Rauf *et al.*, 2008; Selvakumaran *et al.*, 2011) and recently 2-cyclohexan-1-iminium thiocyanate (Salem *et al.*, 2012). The *para*-anilinium cation is planar except the hydrogen atoms of the ammonium moiety. The maximum deviation is 0.002 (2) Å for N1 atom from the least square plane. The thiocyanate ion is linear with N2–C7–S1 bond angle of 179.5 (2)°. The bond lengths and angles are in normal range (Allen *et al.*, 1987; 2002). In the crystal structure, the molecules are linked by the intermolecular hydrogen bonds between the hydrogen atoms of the ammonium moiety with the nitrogen and sulfur atoms of the thiocynato anion (Table 1) to form one-dimensional chain along the *b* axis (Fig. 2).

### Experimental

All solvents and chemicals were of analytical grade and were used without purification. The title compound was prepared by mixing ammonium thiocyanate (0.76 g, 0.01 mol) and *para*-chloroaniline (1.27 g, 0.01 mol) in the presence of HCl. The mixture was refluxed for 1 h. Single crystals were obtained from the solution after one day of evaporation. Yield 85%; m.p: 390.5–393.2 K.

### Refinement

After their location in the difference map, the H-atoms attached to the C were fixed geometrically at ideal positions and allowed to ride on the parent atoms with C—H = 0.93 Å, with *U*_iso_(H)=1.2*U*_eq_(C,) However, the protonated amino hydrogen atoms were located from the Fourier map and refined isotropically.

### Crystal data

**Table:**

C_6_H_7_ClN^+^·NCS^−^	*F*(000) = 768
*M**_r_* = 186.66	*D*_x_ = 1.394 Mg m^−^^3^
Orthorhombic, *P**b**c**a*	Mo *K*α radiation, λ = 0.71073 Å
Hall symbol: -P 2ac 2ab	Cell parameters from 4443 reflections
*a* = 7.743 (2) Å	θ = 1.2–26.5°
*b* = 7.199 (2) Å	µ = 0.60 mm^−^^1^
*c* = 31.913 (10) Å	*T* = 298 K
*V* = 1778.8 (10) Å^3^	Slab, colourless
*Z* = 8	0.50 × 0.43 × 0.30 mm

### Data collection

**Table:**

Bruker SMART APEX CCD area-detector diffractometer	1846 independent reflections
Radiation source: fine-focus sealed tube	1628 reflections with *I* > 2σ(*I*)
Graphite monochromator	*R*_int_ = 0.024
Detector resolution: 83.66 pixels mm^-1^	θ_max_ = 26.5°, θ_min_ = 1.2°
ω scan	*h* = −5→9
Absorption correction: multi-scan (*SADABS*; Bruker, 2000)	*k* = −9→8
*T*_min_ = 0.754, *T*_max_ = 0.841	*l* = −38→40
10422 measured reflections	

### Refinement

**Table:**

Refinement on *F*^2^	Primary atom site location: structure-invariant direct methods
Least-squares matrix: full	Secondary atom site location: difference Fourier map
*R*[*F*^2^ > 2σ(*F*^2^)] = 0.039	Hydrogen site location: inferred from neighbouring sites
*wR*(*F*^2^) = 0.108	H atoms treated by a mixture of independent and constrained refinement
*S* = 1.18	*w* = 1/[σ^2^(*F*_o_^2^) + (0.0501*P*)^2^ + 0.6879*P*] where *P* = (*F*_o_^2^ + 2*F*_c_^2^)/3
1846 reflections	(Δ/σ)_max_ < 0.002
112 parameters	Δρ_max_ = 0.31 e Å^−^^3^
3 restraints	Δρ_min_ = −0.19 e Å^−^^3^

### Special details

**Table:**

Geometry. All e.s.d.'s (except the e.s.d. in the dihedral angle between two l.s. planes) are estimated using the full covariance matrix. The cell e.s.d.'s are taken into account individually in the estimation of e.s.d.'s in distances, angles and torsion angles; correlations between e.s.d.'s in cell parameters are only used when they are defined by crystal symmetry. An approximate (isotropic) treatment of cell e.s.d.'s is used for estimating e.s.d.'s involving l.s. planes.
Refinement. Refinement of *F*^2^ against ALL reflections. The weighted *R*-factor *wR* and goodness of fit *S* are based on *F*^2^, conventional *R*-factors *R* are based on *F*, with *F* set to zero for negative *F*^2^. The threshold expression of *F*^2^ > σ(*F*^2^) is used only for calculating *R*-factors(gt) *etc*. and is not relevant to the choice of reflections for refinement. *R*-factors based on *F*^2^ are statistically about twice as large as those based on *F*, and *R*- factors based on ALL data will be even larger.

### Fractional atomic coordinates and isotropic or equivalent isotropic displacement parameters (Å^2^)

**Table:**

	*x*	*y*	*z*	*U*_iso_*/*U*_eq_	
Cl1	0.41886 (10)	0.01916 (11)	0.749321 (18)	0.0681 (2)	
S1	0.66687 (7)	0.20120 (7)	0.468838 (17)	0.04316 (18)	
N1	0.5614 (2)	0.1442 (3)	0.56935 (6)	0.0404 (4)	
H1A	0.6668 (16)	0.111 (4)	0.5642 (7)	0.048 (7)*	
H1B	0.550 (3)	0.2589 (17)	0.5623 (8)	0.062 (8)*	
H1C	0.490 (3)	0.082 (4)	0.5539 (7)	0.068 (8)*	
N2	0.4214 (2)	0.4537 (3)	0.43930 (7)	0.0510 (5)	
C1	0.4128 (3)	0.2283 (4)	0.63414 (7)	0.0581 (6)	
H1	0.3576	0.3241	0.6199	0.070*	
C2	0.3805 (4)	0.1992 (4)	0.67630 (8)	0.0638 (7)	
H2	0.3038	0.2757	0.6906	0.077*	
C3	0.4620 (3)	0.0576 (3)	0.69664 (6)	0.0446 (5)	
C4	0.5754 (3)	−0.0562 (3)	0.67624 (7)	0.0561 (6)	
H4	0.6301	−0.1524	0.6904	0.067*	
C5	0.6076 (3)	−0.0262 (3)	0.63414 (7)	0.0526 (6)	
H5	0.6847	−0.1023	0.6198	0.063*	
C6	0.5263 (2)	0.1152 (3)	0.61366 (6)	0.0363 (4)	
C7	0.5235 (3)	0.3494 (3)	0.45172 (6)	0.0367 (4)	

### Atomic displacement parameters (Å^2^)

**Table:**

	*U*^11^	*U*^22^	*U*^33^	*U*^12^	*U*^13^	*U*^23^
Cl1	0.0875 (5)	0.0781 (5)	0.0387 (3)	−0.0028 (4)	0.0118 (3)	0.0012 (3)
S1	0.0390 (3)	0.0415 (3)	0.0490 (3)	0.0027 (2)	0.0001 (2)	0.0058 (2)
N1	0.0408 (10)	0.0419 (10)	0.0387 (9)	−0.0012 (8)	−0.0013 (8)	0.0028 (7)
N2	0.0441 (10)	0.0450 (10)	0.0639 (12)	0.0019 (9)	−0.0057 (9)	0.0067 (9)
C1	0.0664 (16)	0.0605 (14)	0.0473 (13)	0.0269 (13)	−0.0001 (11)	0.0027 (11)
C2	0.0710 (16)	0.0712 (16)	0.0491 (13)	0.0305 (14)	0.0105 (12)	−0.0039 (12)
C3	0.0497 (12)	0.0505 (12)	0.0338 (9)	−0.0063 (10)	0.0016 (9)	−0.0028 (9)
C4	0.0690 (16)	0.0528 (13)	0.0465 (12)	0.0168 (12)	0.0043 (11)	0.0104 (10)
C5	0.0606 (14)	0.0531 (13)	0.0440 (12)	0.0189 (11)	0.0098 (10)	0.0047 (10)
C6	0.0354 (9)	0.0366 (10)	0.0368 (10)	−0.0023 (8)	−0.0018 (8)	−0.0012 (7)
C7	0.0372 (10)	0.0338 (9)	0.0392 (10)	−0.0066 (8)	0.0024 (8)	−0.0005 (8)

### Geometric parameters (Å, º)

**Table:**

Cl1—C3	1.736 (2)	C1—H1	0.9300
S1—C7	1.634 (2)	C2—C3	1.363 (3)
N1—C6	1.455 (3)	C2—H2	0.9300
N1—H1A	0.866 (10)	C3—C4	1.365 (3)
N1—H1B	0.860 (10)	C4—C5	1.384 (3)
N1—H1C	0.868 (10)	C4—H4	0.9300
N2—C7	1.160 (3)	C5—C6	1.364 (3)
C1—C6	1.365 (3)	C5—H5	0.9300
C1—C2	1.385 (4)		
			
C6—N1—H1A	108.8 (16)	C2—C3—Cl1	119.39 (18)
C6—N1—H1B	111.9 (19)	C4—C3—Cl1	119.33 (18)
H1A—N1—H1B	108 (3)	C3—C4—C5	119.0 (2)
C6—N1—H1C	110.9 (19)	C3—C4—H4	120.5
H1A—N1—H1C	111 (3)	C5—C4—H4	120.5
H1B—N1—H1C	107 (3)	C6—C5—C4	119.9 (2)
C6—C1—C2	119.4 (2)	C6—C5—H5	120.1
C6—C1—H1	120.3	C4—C5—H5	120.1
C2—C1—H1	120.3	C5—C6—C1	120.9 (2)
C3—C2—C1	119.5 (2)	C5—C6—N1	119.10 (18)
C3—C2—H2	120.3	C1—C6—N1	120.02 (19)
C1—C2—H2	120.3	N2—C7—S1	179.5 (2)
C2—C3—C4	121.3 (2)		
			
C6—C1—C2—C3	−0.3 (4)	C3—C4—C5—C6	−0.1 (4)
C1—C2—C3—C4	0.2 (4)	C4—C5—C6—C1	0.0 (4)
C1—C2—C3—Cl1	−179.0 (2)	C4—C5—C6—N1	−179.8 (2)
C2—C3—C4—C5	0.1 (4)	C2—C1—C6—C5	0.2 (4)
Cl1—C3—C4—C5	179.2 (2)	C2—C1—C6—N1	180.0 (2)

### Hydrogen-bond geometry (Å, º)

**Table:**

*D*—H···*A*	*D*—H	H···*A*	*D*···*A*	*D*—H···*A*
N1—H1*A*···N2^i^	0.87 (2)	2.03 (1)	2.888 (2)	172 (2)
N1—H1*B*···N2^ii^	0.86 (1)	2.08 (1)	2.911 (3)	162 (2)
N1—H1*C*···S1^iii^	0.87 (2)	2.48 (3)	3.285 (2)	155 (2)

Symmetry codes: (i) *x*+1/2, −*y*+1/2, −*z*+1; (ii) −*x*+1, −*y*+1, −*z*+1; (iii) −*x*+1, −*y*, −*z*+1.
